# Developmental Validation of a novel 5 dye Y-STR System comprising the 27 YfilerPlus loci

**DOI:** 10.1038/srep29557

**Published:** 2016-07-13

**Authors:** Rufeng Bai, Yaju Liu, Zheng Li, Haiying Jin, Qinghua Tian, Meisen Shi, Shuhua Ma

**Affiliations:** 1 Department of Radiology, First Affiliated Hospital, Medical College of Shantou University, Shantou 515041, P.R. China; 2 Key Laboratory of Evidence Law and Forensic Science, Ministry of Education, Beijing 100088, P.R. China; 3 Institute of Criminal Sciences and Technology, Municipal Public Security Bureau of Xuchang, Xuchang 461000, P.R. China; 4 Health GeneTech, Ningbo 315000, P.R. China; 5 Collaborative Innovation Center of Judicial Civilization, Beijing 100088, P.R. China.

## Abstract

In this study, a new STRtyper-27 system, including the same Yfiler Plus loci (DYS19, DYS389I, DYS389II, DYS390, DYS391, DYS392, DYS393, DYS385a/b, DYS437, DYS438, DYS439, DYS448, DYS456, DYS458, DYS635, Y-GATA H4, DYS449, DYS460, DYS481, DYS518, DYS533, DYS570, DYS576, DYS627 and DYF387S1a/b), was established using a set of 5 fluorescent dye labels. Primers, internal size standard, allelic ladders and matrix standard set were designed and created in-house for this multiplex system. This paper describes the validation studies conducted with the STRtyper-27Y system using a 3130XL genetic analyzer for fragment length detection that included the analysis of the following parameters and aspects: sensitivity, species specificity, inhibition, haplotype concordance, precision, stutter, DNA mixtures, and stability studies with crime scene samples. The studies demonstrated, that the STRtyper-27Y system provided equivalent overall performance comparable to the latest Yfiler Plus kit, but with enhanced compatibility in terms of instrument platforms and software allowing forensic laboratories to conduct its forensic application and evaluate its performance, all in their own 5 dye Y-STR chemistry system /environment without software or instrument upgrades.

Y-specific short tandem repeat (Y-STR) markers are haploidly inherited in a paternal lineage, and these properties make Y-STRs a useful tool in investigations of sexual assault, paternity and genealogical tests and evolutionary studies^1^,^2^,^3^. To date, 17 Y-STR loci included in the Yfiler PCR amplification kit used in forensics have adequate resolution of different paternal lineages in many populations, but fail to differentiate between related males who belong to the same paternal lineage or to separate paternal lineages in populations expressing low Y-chromosome diversity^4^. In light of the continuous demand for developing more efficient and discriminative typing systems, new commercial STR kits with expanded set of Y-STR markers have been produced in the last few years. 2013, Life Technologies launched an expanded and improved version of Yfiler kit, the Yfiler Plus kit, a 6-dye multiplex system which combine 17 Yfiler loci (DYS19, DYS385a/b, DYS389I/II, DYS390, DYS391, DYS392, DYS393, DYS437, DY438, DYS439, DYS448, DYS456, DYS458, DYS635 and Y-GATA-H4), three new Y-STRs (DYS460, DYS481 and DYS533), plus seven rapidly mutating (RM) Y-STRs^5^ (DYS449, DYS518, DYS570, DYS576, DYS627 and DYSF387S1a/b) by taking advantage of the performance and manufacturing improvements applied to Applied Biosystems Next Generation STR systems. Several validation studies have proved the Yfiler Plus kit yield high resolution paternal lineage differentiation and provide a considerable improvement compared to Yfiler kit^6^,^7^,^8^,^9^. In this study, we developed a new 5-dye STRtyper-27 system containing the same Y-STR loci included in the Yfiler Plus system using existing instrument platforms and software. Also, developmental validation studies of sensitivity, species specificity, inhibition, haplotype concordance, precision, stutter, DNA mixtures, and stability studies with crime scene samples were applied following “SWGDAM Guidelines for Validation of Probabilistic Genotyping Systems (final approval on 06/15/2015)” and the Chinese National Standard (CNS) “Basic Quality Requirements of Forensic Science Human Fluorescent STR Multiplex PCR Testing Reagent” (GA/T815-2009). The results showed that the STRtyper-27 system with equivalent overall performance comparable to the latest Yfiler Plus kit is economic, rapid and robust for forensic application in Chinese population.

## Methods

### Ethics statement and Quality control

The main experiments were performed in the Key Laboratory of Evidence Law and Forensic Science, Ministry of Education, P.R. China which is accredited according to the ISO 17025 standard. This study was conducted according to the humane and ethical research principles and approved by the Ethical committee of Shantou Medical College, P.R. China. Before getting involved in the study, all the participants signed the written informed consents for the human blood sample collections and subsequent analyses. Animal blood samples and microbial DNA isolates were approved and prepared from the Institute of Microbiology, Chinese Academy of Sciences, P.R. China.

### Primer design and multiplex amplification

The STRtyper-27 system encompasses all Yfiler Plus loci (DYS19, DYS385a/b, DYS389I/II, DYS390, DYS391, DYS392, DYS393, DYS437, DY438, DYS439, DYS448, DYS456, DYS458, DYS635, Y-GATA-H4, DYS460, DYS481, DYS533, DYS449, DYS518, DYS570, DYS576, DYS627 and DYSF387S1a/b). Eight among the chosen markers (DYS456, DYS576, DYS458, DYS460, DYS393, DYS391, DYS438, and DYS389I) were designed specifically for amplicons smaller than 220 bp for the detection of degraded DNA samples. All the primers were designed for this study using the Primer3 program (http://bioinfo.ut.ee/primer3–0.4.0/primer3/input.htm). Each primer was checked for potential structures of the self dimmer using the AutoDimer v1.1 software and non-specific hybridizations in other genome regions using NCBI Basic Local Alignment Search Tool (BLAST). The four dyes used in the STRtyper-27 system to label primers are 6 FAM(blue), HEX(green), TAMRA(yellow), and ROX(red) dyes(Life Technologies). The fifth dye, SIZE-500 (orange)(Health GeneTech, China), was used to label the internal size standard. 27 loci were organized by expected amplicon size and assigned to the first four different fluorescent dyes in order to achieve an evenly balanced genotyping assay for a single PCR and electrophoretic separation. For details on the marker’s repeat structure, genomic location, final concentration, observed allelic ranges, haplotypes of control DNA 9948, 007 and dye labeling refer to the Table 1.

The validation was performed with both 10 μL and 25 μL reaction systems, respectively. The 10 μL reaction volume contained: 5.0 μL PCR Master Mix, 2.5 μL Primer Mix and 0.2~2 ng of template DNA. The 25 μL reaction volume contained: 12.5 μL PCR Master Mix, 6.25 μL Primer Mix and 0.2~2 ng of template DNA. The PCR Master Mix included DMSO 10 mM, 125 mM Tris buffer, 125 mM KCl, and 65 mM (NH4)_2_SO_4_. The Primer Mix included appropriate concentration of primers, 7.5 mM MgCl_2_, 7.5 mM dNTPs, 2.5 mg/mL bovine serum albumin (BSA), and 5 U/μL High Specificity *Taq* DNA polymerase (Dongsheng BioTech, China). The optimized formulation of the Master Mix and Primer Mix improve performance and support the direct amplification of a larger number of Y-STR markers for both casework and single-source samples.

Amplification was performed in GeneAmp 9700 thermal cyclers (Life Technologies) and required 5 min of initial denaturation at 95 °C. Subsequent cycles of denaturation at 94 °C for 10 sec, annealing at 61 °C for 1 min and elongation at 70 °C for 30 sec were repeated 28~30 times according to DNA sample types, followed by 20 min of the final elongation at 60 °C to avoid splitting peaks.

### Detection and genotyping

The Applied Biosystems 3130XL Genetic Analyzer (Life Technologies) was set with Dye set G5 to process the data from the five dyes 6 FAM (blue), HEX (green), TAMRA (yellow), ROX (red) and SIZE-500 (orange) after an appropriate matrix. Samples for capillary electrophoresis were prepared by mixing 1 μL of PCR product, with 9 μL of a 17:1 Hi-Di Formamide (Life Technologies) and SIZE-500 internal size standard (Health GeneTech). All prepared samples were separated on the ABI 3130XL Genetic Analyzer using 36 cm capillaries (Life Technologies). Standard run parameters involved: sample injection for 10 s at 3 kV and electrophoresis at 15 kV at 60 °C in POP-4 polymer (Life Technologies) as indicated in the HIDFragmentAnalysis36_POP4_1 module. All genotyping was performed with GeneMapper *ID v.3.2.1* software (Life Technologies) with in-house allelic ladder, programmed Panel and Bin sets for each marker. All alleles present in the allelic ladder were sequenced to confirm length and repeat unit structure, using Big Dye Terminator v.3.1 chemistry (Life Technologies). Positive control DNA of 9948 (Promega Corporation) and 007 (Life Technologies) human cell line samples were used as positive samples in the electrophoresis. The nomenclature used was that of the latest recommendations for the DNA commission of the International Society of Forensic Genetics^10^.

### Sensitivity, species specificity, and inhibition studies

To evaluate the sensitivity of this STRtyper-27 system, a serial dilutions of positive control DNA 9948 were analyzed in triplicate with quantities from 4 ng to 62.5 pg per reaction.

Assessing species specificity encompassed testing performance of the assay in amplifying 10 ng of non-human DNA from common male animal species (horse, dog, pig, cow, sheep, chick, duck, cat, fish, rabbit and mouse) and microorganisms pool (*Escherichia coli, Micrococcus luteus, and Streptococcus salivarius*). Three replicates for each species and microorganisms listed were tested.

Resistance to inhibition was assessed using 4 most common inhibitors encountered in our practice-hematin, humic acid, tannic acid, and calcium with the following various concentrations-hematin (5 μM, 25 μM, 50 μM, 75 μM, and 100 μM), humic acid (10 ng/μL, 20 ng/μL, 30 ng/μL, 50 ng/μL, 70 ng/μL and 90 ng/μL), tannic acid (50 ng/μL, 100 ng/μL, 150 ng/μL, 200 ng/μL, 300 ng/μL, 500 ng/μL), and calcium 0.2 mM, 0.4 mM, 0.8 mM, 1.0 mM, 2.0 mM, 3.0 mM), respectively. All tests on sensitivity, specificity and inhibition employed a 50 RFU analytical threshold on the ABI 3130 XL platform.

### Concordance, precision and stutter calculations

A total of 1225 sample already typed for Yfiler Plus kit in a previous haplotype report [YHRD accession number: Henan, China[Han], YA004057]^11^ were processed on the STRtyper-27 system. The results referring to the same 27 Y-STRs were compared for haplotype concordance.

A subset sample profiles from the concordance study were also used to measure the deviation of each sample allele size from the corresponding allele size in the allelic ladder and to calculate stutter. Peaks one repeat smaller or larger than the true allele (±0.5 bp) were determined to be stutter peaks. The proportion of stutter product relative to the main allele (percent stutter) was measured by dividing the height of the stutter peak by the height of the associated allele peak^12^. In this study, the analytical threshold was lowered to 10 RFU and the stutter filters were set to 1% to detect the stutter peak heights.

### Mixture study

Evidence samples are frequently composed of more than one individual’s DNA. For correct interpretation of results from mixtures, it is important to know the limit of the minor contributing component that can be resolved. A mixed male/male DNA sample (9948 and 007) with known ratios (19:1, 18:2, 16:4, 14:6, 12:8, 1:1, 8:12, 6:14, 4:16, 2:18, 1:19) for a total of 1 ng of DNA were tested in triplicate. Mixed female/male DNA samples with known ratios (9947 and 9948 mixed at 1000:1, 100:1, 10:1 and 1:1) were also checked the ability of STRtyper-27 system to amplify male DNA in the presence of an excess of female material. The amount of male DNA was kept constant at 125 pg, while amounts of female DNA were varied (125 ng, 12.5 ng, 1.25 ng, and 125 pg).

### Stability study

Crime scene samples were examined to evaluate the performance of the STRtyper-27 system, which are 15 bloodstains on different matrices, 4 saliva stains on toothbrush, denim fabric, cigarette and on bottleneck, 5 semen/female vaginal secretion mixtures, 5 muscle tissues, 5old bones, 6 hair samples, and 4 formalin fixed and paraffin embedded biopsy tissues (FFPEB). The age of the samples ranged from 10 days up to 60 months. All samples were previously quantified using Quantifiler Duo Human DNA quantification Kit (Thermo Fisher) and analyzed with Yfiler Plus kit, respectively.

### Statistics

Haplotype frequencies were determined using the counting method. Haplotype Diversity (HD) was calculated using the formula HD = *n(1* − Σ*p*_*i*_^2^*)/(n* − *1)*, following Nei^13^, where *n* and *p*_*i*_ denote the total number of samples and the relative frequency of the *i*-th allele, respectively. The discrimination capacity (DC) of the marker systems included: Yfiler haplotype (Yfiler = DYS19, DYS389I, DYS389II, DYS390, DYS391, DYS392, DYS393, DYS437, DYS438, DYS439, DYS385, DYS448, DYS456, DYS458, DYS635, YGATAH4), and 27 Y-STR haplotype (=Yfiler + DYF387S1, DYS449, DYS627, DYS576, DYS570, DYS533, DYS518, DYS481 and DYS460). The discriminatory capacity was determined by dividing the number of different haplotypes by the number of samples in that population^14^. For all analyses the DYS385 and DYF387S1 locus were treated as a single haplotype and not two separate alleles.

## Results and Discussion

### Optimization of the STRtyper-27 typing system

**Figure 1:**
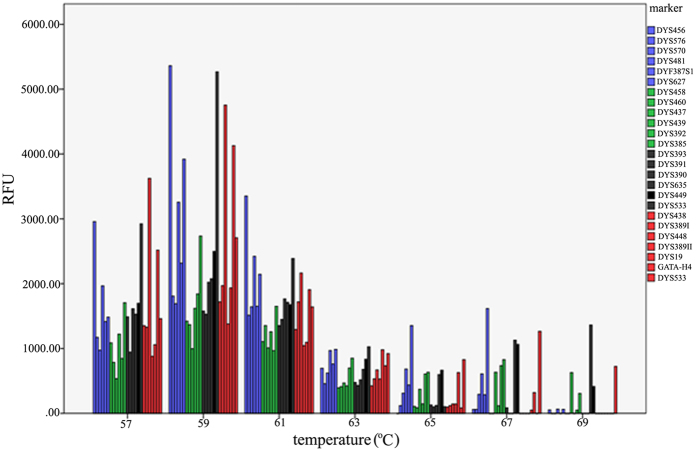
Variations of amplification efficiency at various PCR annealing temperatures. Seven annealing temperatures were tested: 57 °C, 59 °C, 61 °C, 63 °C, 65 °C, 67 °C and 69 °C. Amplification of 1 ng 9948 DNA was performed for 30 cycles on Applied Biosystems GeneAmp 9600, and 1 μl of each reaction was simultaneously analyzed on an Applied Biosystems 3130XL Genetic Analyzer with a 3 kV, 10 s injection.

A new STRtyper-27 multiplex system that allows co-amplification of the same 27 Y-STR loci included in the Yfiler Plus kit was developed and detected by existing instrument platforms and software so that forensic laboratories can conduct experiments, evaluate its performance, and compare results to the latest commercial available Y-STR kit, all in their own 5-dye Y-STR chemical environment. During the optimization process of PCR conditions, annealing temperatures between 57 °C and 69 °C were applied to the amplification of 1 ng positive control DNA 9948. The nonspecific peaks were relatively obvious at the annealing temperature of 57 °C. Full and clean profiles were obtained when PCR was performed with annealing temperatures between 59 °C and 63 °C, and fragment dropouts were observed at the annealing temperature of 65 °C (Supplementary Fig. S1). The best amplification profile was obtained at the annealing temperature of 61 °C (Fig. 1). Both 10 μL reaction system and 25 μL reaction system showed the same results.

Altering the number of PCR cycles can be used to adapt the reaction conditions to varying DNA template concentrations. First, we tested the low-copy-number DNA, 125 pg, 62.5 pg, and 31.25 pg positive control DNA 9948, with cycle numbers of 28, 29, and 30. As expected, in both 10 μL and 25 μL reaction system, the signal intensities of called alleles increase with higher cycle numbers. Afterwards, we tested the high-copy-number DNA, 10 ng and 20 ng positive control DNA 9948, using both 10 μL and 25 μL reaction systems. Robust amplifications and full profiles were obtained with cycle numbers of 28, 29, and 30 using a threshold of 50 RFU for allele calling. However, 30 cycle amplifications of 20 ng DNA gave rise to pull-up peaks when applying PCR products to analysis without prior dilution (data not shown).

**Figure 2:**
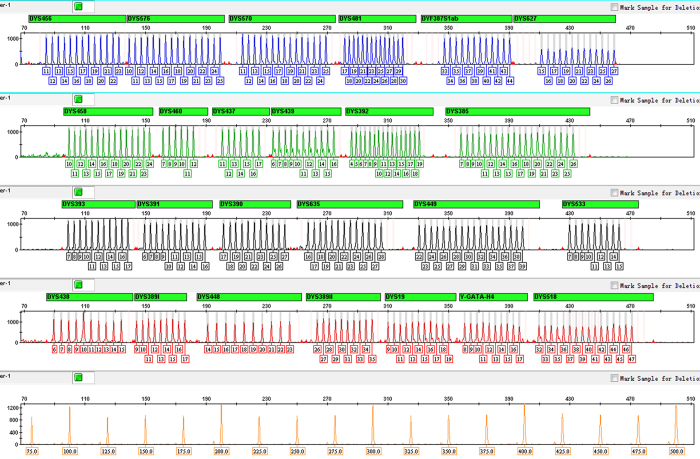
Electropherogram of allelic ladders and internal size standard in the STRtyper-27 system. The four dye panels for allelic ladders correspond to (from top to bottom) 6FAM (blue), HEX (green), TAMRA (yellow), ROX (red) dye-labeled peaks. The haplotype is shown with the allele number displayed underneath each peak. The fifth panel reserved for internal size standard labels an orange dye: SIZE500 (a total of eighteen fragments: 75, 100, 125, 150, 175, 200, 225, 250, 275, 300, 325, 350, 375, 400, 425, 450, 475 and 500 bp).

After the optimization of the multiplex, 27 Y-STRs were successfully amplified in a single PCR reaction. The system produced peak height balanced haplotype results with amplicon sizes of 80–478 bp. Average peak heights ranged from 700 to 6000 RFUs, and balance between peaks amplified by the same pair of primers such as DYS385 or DYF387S1 was >70%. The profile of allelic ladder and control DNA 9948 were presented in Fig. 2 and Supplementary Fig. S2, respectively.

### Sensitivity, species specificity, and inhibition studies

**Figure 3:**
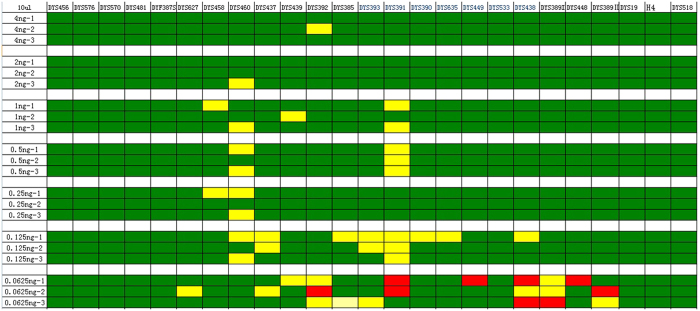
The sensitivity of the 10 μL system. Green represents peak height ratio > 60% within the same color. Yellow represents peak height ratio < 60% within the same color. Red represents allele drop-out.

**Figure 4:**
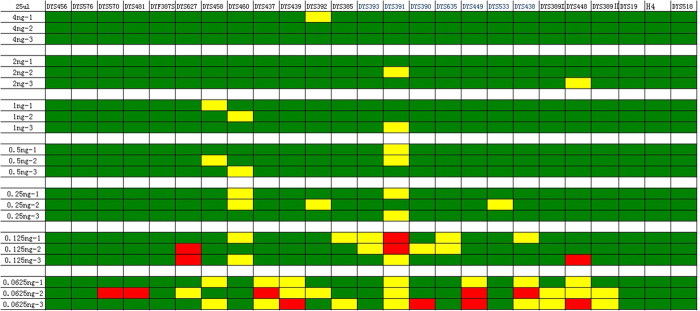
The sensitivity of the 25 μL system. Green represents peak height ratio > 60% within the same color. Yellow represents peak height ratio < 60% within the same color. Red represents allele drop-out.

A novel system developed for forensic application needs to be able to produce a profile with sub-nanogram quantities of template DNA. To test the sensitivity of the STRtyper-27 system, positive control DNA 9948 was serially diluted from 4 ng to 62.5 pg per reaction. In the 10 μL reaction system, occasional allele dropouts were found when ≤62.5 pg DNA was used as the template (Fig. 3), whereas in the 25 μL reaction system, occasional allele dropouts were found when ≤125 pg DNA was used as the template (Fig. 4). As expected, the number of dropouts increases with decreasing DNA concentrations. Therefore, for severely degraded DNA samples, DNA template amount higher than 1 ng is recommended for reliable results.

No reproducible signal or characteristic human profiles could be obtained above 50 RFUs threshold for all of the domestic species and microbial pool genomic DNA samples tested demonstrating the low cross-reactivity with non-human species of the STRtyper-27 system (Supplementary Fig. S3).

An inhibition study was also performed with hematin, humic acid, tannic acid, and calcium, respectively, added directly to the STR reaction to test the robustness of the STRtyper-27 system. For hematin the range of examined concentrations was 5–100 μM with 50 μM being the upper resistance value allowing successful amplification across all 27 loci (Supplementary Fig. S4). This result is much better than the one reported for Yfiler, which exhibits overall inhibition if hematin concentration exceeds 16 μM^15^. In case of humic acid, we applied 1–70 ng/μL of inhibitor and observed non-problematic amplification up to the addition of 20 ng/μL of humic acid. Full profiles were obtained with 50 ng/μL tannic acid, partial profiles of locus drop-out observed with higher amplicon lengths at the 100 ng/μL to 150 ng/μL tannic acid, and no profiles were observed at 200 ng/μL tannic acid. Lastly, full profiles were obtained with 0.2 mM calcium, partial profiles at the 0.4 to 1.0 mM calcium and no profiles were observed above 2.0 mM calcium. Results demonstrated the STRtyper-27 system could tolerate considerable concentrations of inhibitors.

### Concordance, precision, and stutter calculations

All profiles from 1225 samples using the STRtyper-27 system were concordant with the Yfiler Plus kit reference profiles run on the required 3500 instrument and software. By comparison, the genotyping results of the same 27 Y-STRs were identical (data not shown). We compared the haplotype resolution of combined Y-STRs. 961 haplotypes were observed unique and the discrimination capacity of the 17 Y-STR loci was 86.94% with 1065 different haplotypes. By the addition of 10 Y-STRs (DYS449, DYS460, DYS481, DYS518, DYS533, DYS570, DYS576, DYS627 and DYSF387S1a/b), an improved discrimination capacity was obtained as 98.94% from 1212 observed haplotypes in 1225 Henan Han samples, indicating that the discrimination power of 27 Y-STR haplotypes in Henan Han is high for forensic and kinship casework.

Determining the sizing precision includes evaluation of measurement error and assessing the performance for accurate and reliable genotyping^12^. A subset of 200 extracted DNA and directed samples from the concordance study were used to measure the deviation of each sample allele from the corresponding allele size in the allelic ladder. All sample alleles tested were within ± 0.5 bp of the corresponding alleles in the allelic ladder demonstrating appropriate precision for sizing microvariants that differ by a single base (Supplementary Fig. S5).

Stutter is characterized by the presence of an allelic-like signal that is typically one repeat shorter than the parent peak and is significantly less intense than the parent peak (<20%)^16^. It is important to distinguish between stutter and a true allele in order to resolve mixtures of DNA. In this study, the analytical threshold of minimum stutter peak height was set to 10 RFUs for the accuracy of stutter file for GeneMapper ID *v3.2.1.* In Supplementary Table S1 the relative intensities of stutter signals are shown for each of the 27 Y-STR locus. The average stutter intensity of all loci tested in this study was 9.04 ± 2.8%. According to previous developmental validation study^17^, the percent stutter is usually more pronounced for shorter repeat motifs and generally increases with allele length. In this study the tri-nucleotide repeat locus DYS481 displayed the highest relative stutter average peaks at 18.19% of the main allele. The locus DYS449 and DYS518, which both contain longer allele ranges, displayed the higher relative stutter peaks at 15.70% and 14.42% of the main allele, respectively. The stutter range observed is similar to that seen with autosomal STRs and consequently stutter signals should normally be distinguishable from allele signals.

### Mixture study

**Figure 5:**
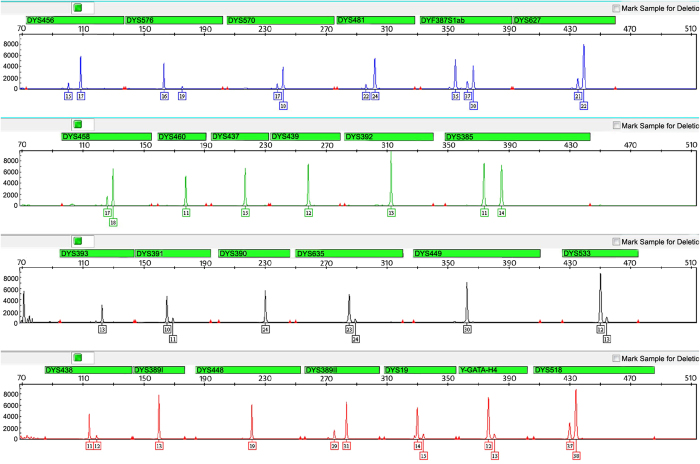
Electropherogram of a male/male DNA mixture sample (9948/007) at ratio of 19:1 with a total of 1 ng of DNA. The alleles attributable to the minor component, even when the major component shares an allele, are highlighted.

To evaluate male/male mixture detection performance, positive control DNA 9948 and 007 in ratios of 19:1, 18:2, 16:4, 14:6, 12:8, 1:1, 8:12, 6:14, 4:16, 2:18, 1:19, were distributed and amplified for a total of 1 ng of mixed DNA. Alleles unique to the minor contributor were counted and presented as a percentage of the total number of unique allel expected (Supplementary Table S2). When the mixture ratio was increased to 18:2 and 2:18, an average of 88%, 77%, respectively, of the minor alleles was detected. And when the mixture ratio was increased to 19:1 and 1:19, an average of 51%, 37% respectively, of the minor alleles was called. The profile of 9948/007 DNA with ratio of 19:1 was showed in Fig. 5. The results showed that as the mixture ratio increased, the percentage of minor alleles detected decreased.

For the detection of female/male mixture, with known ratios (9947 and 9948 mixed at 1000:1, 100:1, 20:1 and 1:1), a series of samples were prepared in which 125 pg of 9948 DNA was admixed with varying quantities of 9947 DNA and the total admixture amplified in a single reaction. Full profiles were obtained when the male DNA component comprised 1/1 th, 1/10 th, 1/100 th, and 1/1000 th of the total. The results demonstrate that even in the presence of an excess of female DNA, it is possible to obtain a full profile of the male contributor with the STRtyper-27 system.

### Stability study

The STRtyper-27 system was also tested with DNA extracts obtained from crime scene samples such as blood, saliva, semen stains, and from different tissues (including hairs, skin, muscle, old bone and formalin fixed and paraffin embedded biopsy) to evaluate the effect of biological sample types and quantity, PCR inhibitors from background contaminants, and DNA degradation and the performance to obtain full or partial profiles compared with Yfiler Plus kit.

The STRtyper-27 typing results obtained from 24 biological stains (blood, saliva, and semen) showed full allelic concordance with the reference profiles previously obtained using Yfiler Plus kit. No allelic or locus drop out was observed when 500 pg of DNA was amplified, and several samples with less than 200 pg yielded full profiles. Degraded DNA obtained from 10 old bone and muscle samples (postmortem interval about 3–60 months), 6 hair roots and 4 FFPEB tissues were used to challenge the STRtyper-27 system against DNA degradation, in parallel with the Yfiler Plus kit. Seven degraded DNA samples rendered both full concordant Y-STR profiles or significant partial Y-STR profiles with both STRtyper-27 and Yfiler Plus kit. Supplementary Fig. S6 shown the STRtyper-27 electropherograms obtained from one degraded DNA bone sample.

## Conclusion

This article outlines the development of a STRtyper-27 multiplex system that incorporates all loci of the latest available Yfiler Plus kit. Eight Y-STR loci (DYS456, DYS576, DYS458, DYS460, DYS393, DYS391, DYS438 and DYS389I) are less than 220 bp in length in this multiplex, helping to ensure that full profiles can be obtained with degraded DNA samples. The developmental validation studies demonstrated that the STRtyper-27 system generates minimal cross-reactivity, high quality, precise, accurate, and low level sensitive profiling STR data, even from a wide variety of forensic biological samples with sub-nanogram amounts of genomic DNA. The STRtyper-27 system provides an equivalent overall performance to the latest forensic 6-dye Yfiler Plus kit, but with significant compatibility in terms of existing instrument platforms and software that would be cost efficient especially for the local, provincial, and developing national ones.

## Additional Information

**How to cite this article**: Bai, R. *et al*. Developmental Validation of a novel 5 dye Y-STR System comprising the 27 YfilerPlus loci. *Sci. Rep.* **6**, 29557; doi: 10.1038/srep29557 (2016).

## Supplementary Material

Supplementary Information

## Figures and Tables

**Table 1:** Characterization and primer-related information on 27 Y-STR loci present in the multiplex system.

Locus	Physical position	Repeat motif	Concentration [μM]	Allele range	Size range (bp)	Haplotype of 9948	Haplotype of 007	Dye
DYS456*	Yp11.2(4.33Mb)	AGAT	0.2100	11–23	80–136	17	15	6FAM
DYS576*^,★^	Yp11.2(7.05Mb)	AAAG	0.1250	10–25	139–199	16	19	6FAM
DYS570★	Yp11.2(6.86Mb)	TTTC	0.1675	11–25	209–272	18	17	6FAM
DYS481	Yp11.2(8.43Mb)	CTT	0.1650	17–31	278–325	24	22	6FAM
DYF387S1★	Yp11.2(28.0Mb)	AAAG	0.1675	32–44	335–390	35/38	35/37	6FAM
DYS627★	Yp11.2(8.65Mb)	AAAG	0.3350	14–27	395–458	22	21	6FAM
DYS458*	Yp11.2(7.93Mb)	GAAA	0.1350	10–24	99–152	18	17	HEX
DYS460*	Yq11.222(21.05Mb)	ATAG	0.3150	7–12	161–189	11	11	HEX
DYS437	Yq11.21(12.98Mb)	TCTA	0.0750	11–17	195–229	15	15	HEX
DYS439	Yq11.21(13.03Mb)	AGAT	0.1750	6–16	233–278	12	12	HEX
DYS392	Yq11.222(21.04Mb)	TAT	0.3400	4–19	285–333	13	13	HEX
DYS385a/b	Yq11.222(19.26Mb)	GAAA	0.1625	7–26	353–439	11/14	11/14	HEX
DYS393*	Yp11.2(3.19Mb)	AGAT	0.1250	7–17	99–142	13	13	TAMRA
DYS391*	Yq11.21(12.61Mb)	TCTA	0.1500	6–16	145–188	10	11	TAMRA
DYS390	Yq11.221(15.78Mb)	[TCTA] [TCTG]	0.1625	17–27	201–242	24	24	TAMRA
DYS635	Yq11.21(12.89Mb)	TSTA compound	0.3250	16–28	252–313	23	24	TAMRA
DYS449★	Yp11.2(8.28Mb)	TTTC	0.3000	22–39	330–402	30	30	TAMRA
DYS533	Yq11.221(18.39Mb)	ATCT	0.3250	7–15	429–470	12	13	TAMRA
DYS438*	Yq11.21(13.38Mb)	TTTTC	0.3750	6–15	89–139	11	12	ROX
DYS389I*	Yq11.21(13.12Mb)	[TCTG] [TCTA]	1.1500	9–17	143–177	13	13	ROX
DYS448	Yq11.223(22.78Mb)	AGAGAT	0.4500	14–23	190–251	19	19	ROX
DYS389II	Yq11.21(13.12Mb)	[TCTG] [TCTA]	0.6750	26–35	258–304	31	29	ROX
DYS19	Yp11.2(10.13Mb)	TAGA	0.4750	9–19	309–351	14	15	ROX
Y-GATA-H4	Yq11.1(17.25Mb)	TAGA	0.5000	8–18	360–401	12	13	ROX
DYS518★	Yq11.221(17.32Mb)	AAAG	0.5250	32–47	409–478	38	37	ROX

^*^Are labeled for the MiniSTR loci.

^★^Are labeled for the rapidly mutating (RM) Y-STRs.
