# Supplementary material for: Developmental Validation of a novel 5 dye Y-STR System comprising the 27 YfilerPlus loci
Source: Sci Rep. 2016 Jul 13;6:29557. doi: 10.1038/srep29557 (PMC4942765; doi:10.1038/srep29557)
Supplement: Supplementary Information [file srep29557-s1.pdf]

Developmental Validation of a novel 5 dye Y-STR System comprising the 27 YfilerPlus loci

Rufeng Bai[1,2,*], Yaju Liu[3,*], Zheng Li[4], Haiying Jin[4], Qinghua Tian[1], Meisen Shi[1,2], Shuhua Ma[5]

[1]Key Laboratory of Evidence Science (China University of PoliticalScience and Law), Ministry of Education, Beijing 100088, P.R.China; [2] Collaborative Innovation Center of Judicial Civilization, Beijing 100088, P.R.China; [3]Institute of Criminal Sciences and Technology, Municipal Public Security Bureau ofXuchang, Xuchang 461000, P.R.China; [4] HEALTH GeneTech, Ningbo315000, P.R.China; [5]Department of Radiology, First Affiliated Hospital, Medical Collegeof Shantou University, Shantou 515041, P.R.China

*These authors contributed equally to this work

Correspondence and requests for materials should be addressed to Meisen Shi and Shuhua Ma (email: shimeisen2000@163.com, mashuhua6699@163.com)

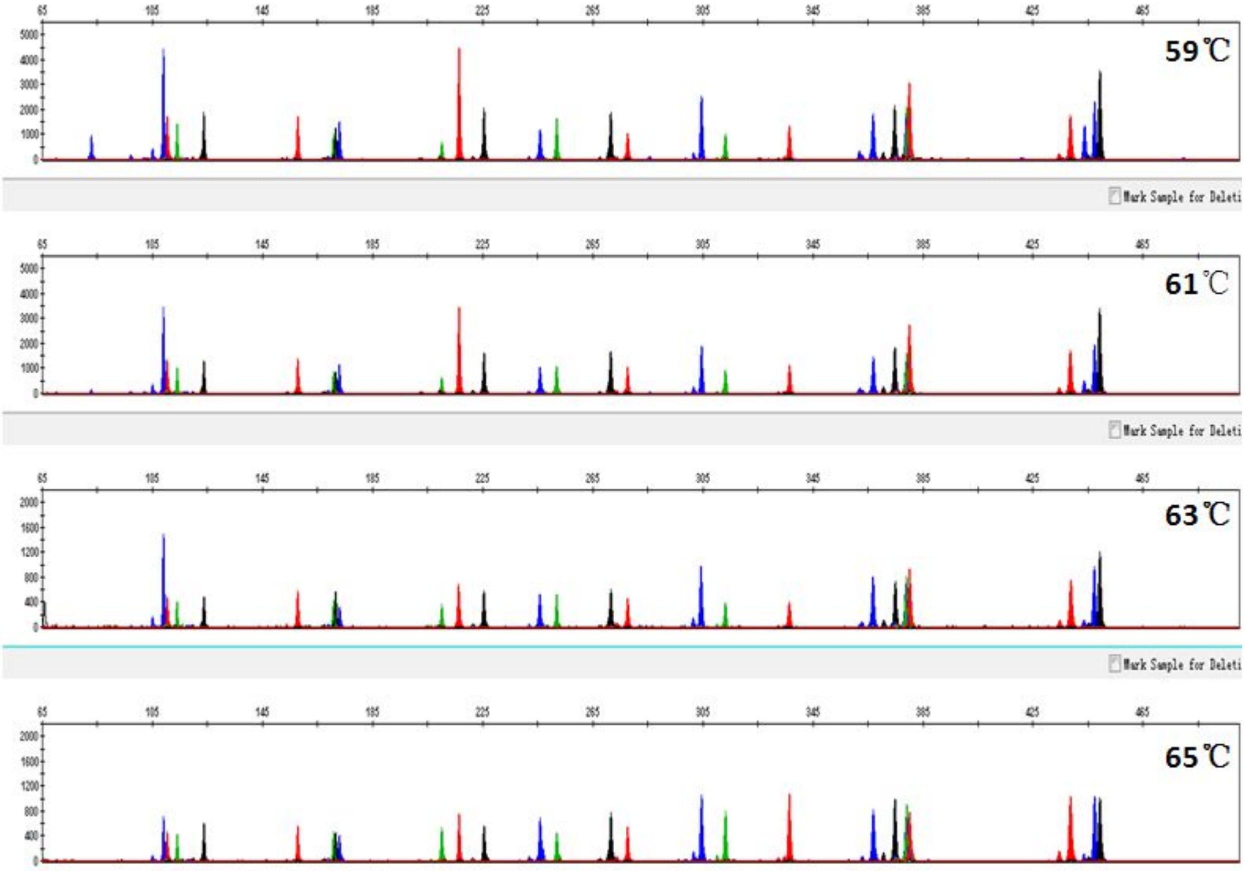

FigureS1. Representative electropherograms for reactions using annealing temperatures from 59°C to 65°C

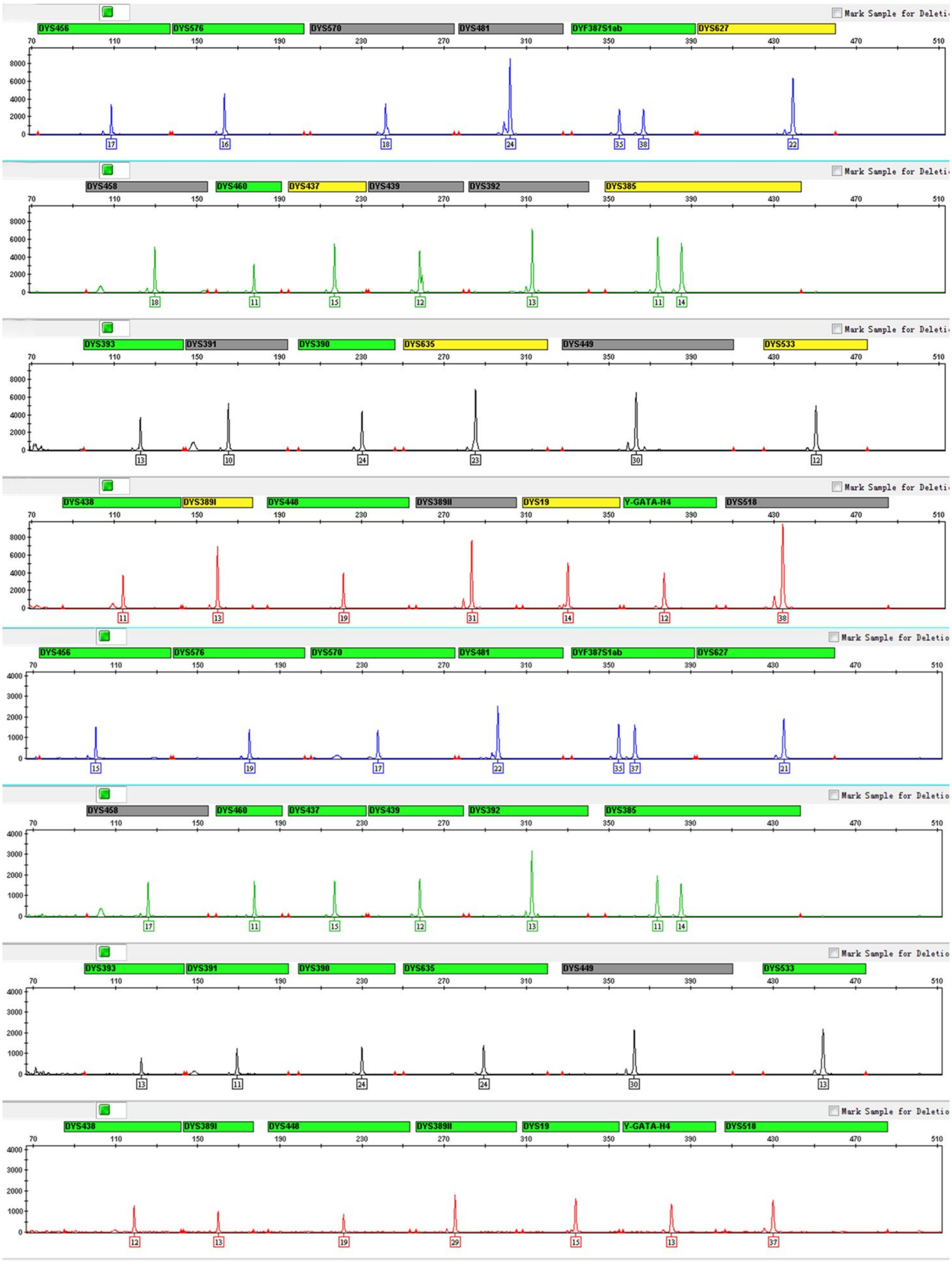

Figure S2.Electropherogram of the Control DNA 9948(upper) and 007(lower) amplified by STRtyper-27 system

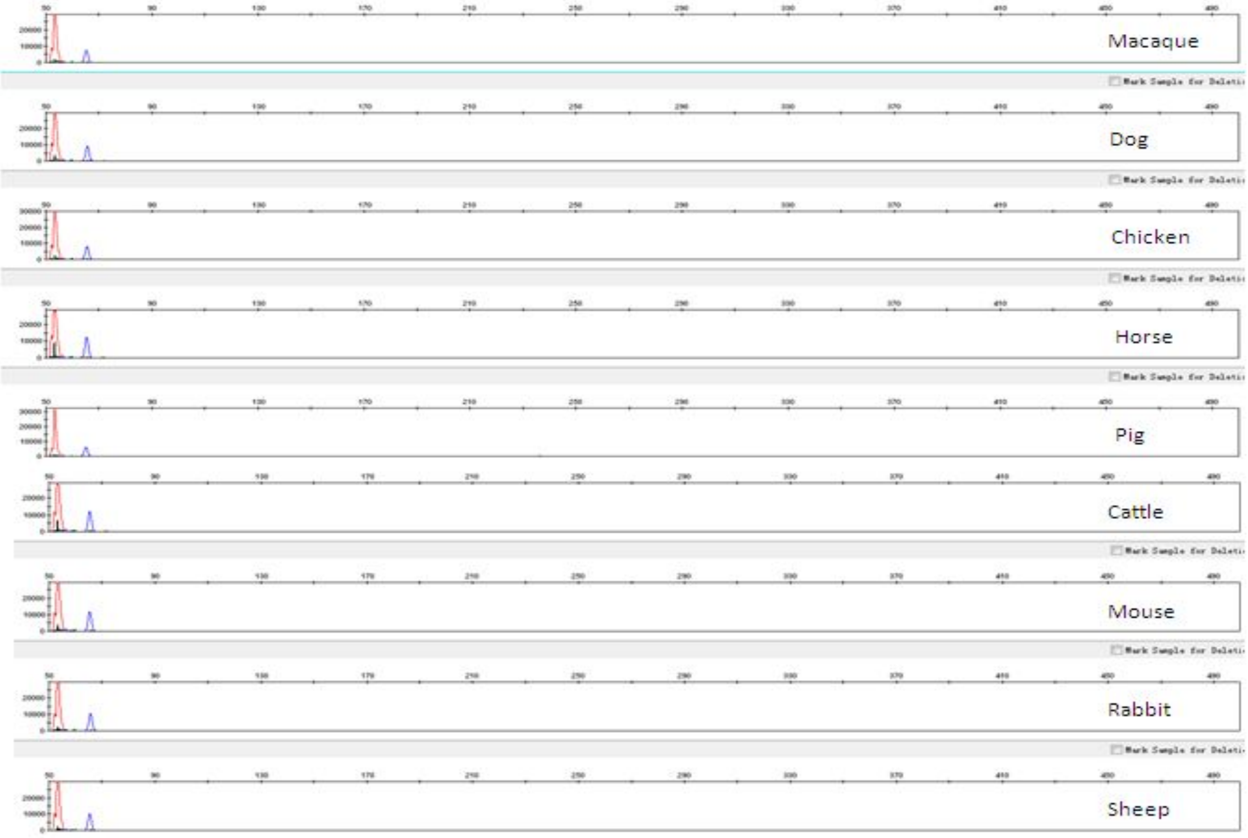

Figure S3.Representative results of the species specificity assessment

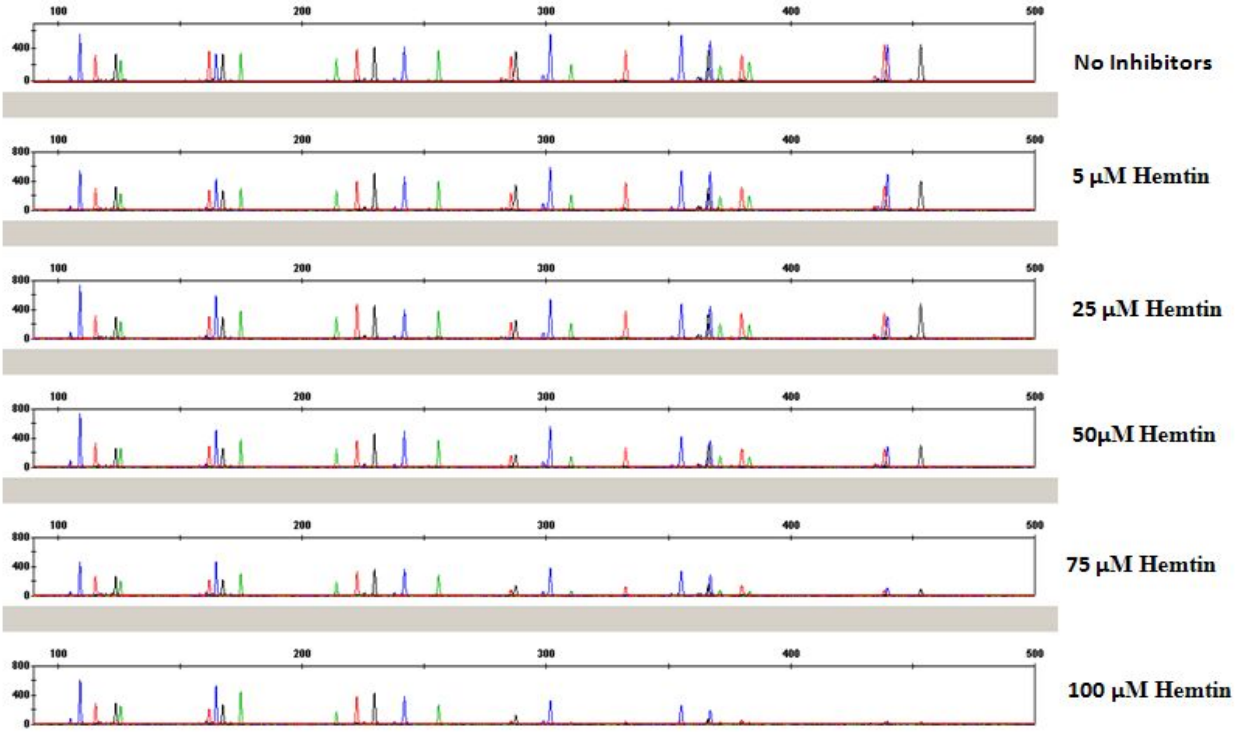

Figure S4. Representative electropherogram of 1ng 9948 amplified in the various concentrations of the hematin in the 25μL system.

Locus	Stutter range(%)	Stutter average(%)	Standard Deviation(%)
DYS456	2.03-14.41	10.32%	1.88%
DYS576	8.20-13.57	10.61%	1.05%
DYS570	6.74-13.48	9.08%	0.97%
DYS481	1.91-23.15	18.19%	1.91%
DYF387S1	4.45-18.28	10.97%	2.32%
DYS627	6.24-11.01	8.57%	1.24%
DYS458	1.80-13.00	10.06%	1.34%
DYS460	3.75-9.62	5.70%	1.31%
DYS437	3.98-9.15	6.40%	1.28%
DYS439	4.76-10.90	6.81%	1.18%
DYS392	3.27-17.06	9.79%	2.38%
DYS385	5.08-14.16	9.14%	2.51%
DYS393	1.21-11.71	7.99%	1.58%
DYS391	5.22-12.97	7.51%	1.04%
DYS390	6.45-12.91	8.89%	1.00%
DYS635	4.49-12.30	7.83%	1.34%
DYS449	11.41-20.79	15.70%	1.81%
DYS533	4.17-7.73	6.06%	0.85%
DYS438	1.46-10.47	4.57%	2.27%
DYS389I	5.35-9.71	6.90%	0.98%
DYS448	2.80-5.43	3.85%	0.76%
DYS389II	10.05-16.18	12.96%	1.20%
DYS19	4.74-8.18	6.31%	0.95%
Y-GATA-H4	5.34-10.28	7.42%	1.04%
DYS518	11.12-21.42	14.42%	2.00%

Table S1 Observed stutter values for 27 Y-STR loci

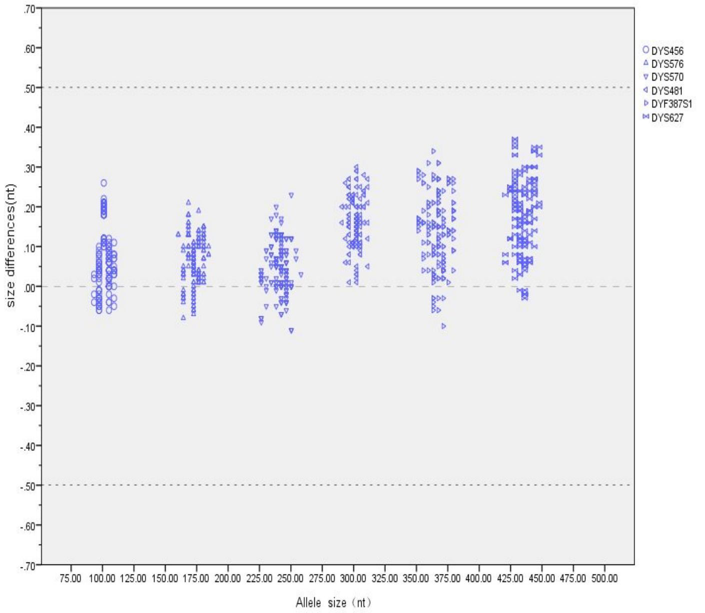

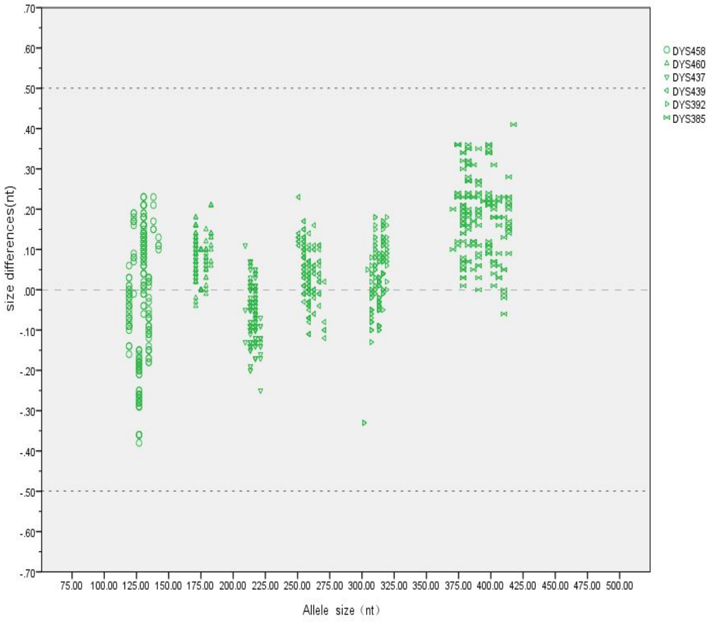

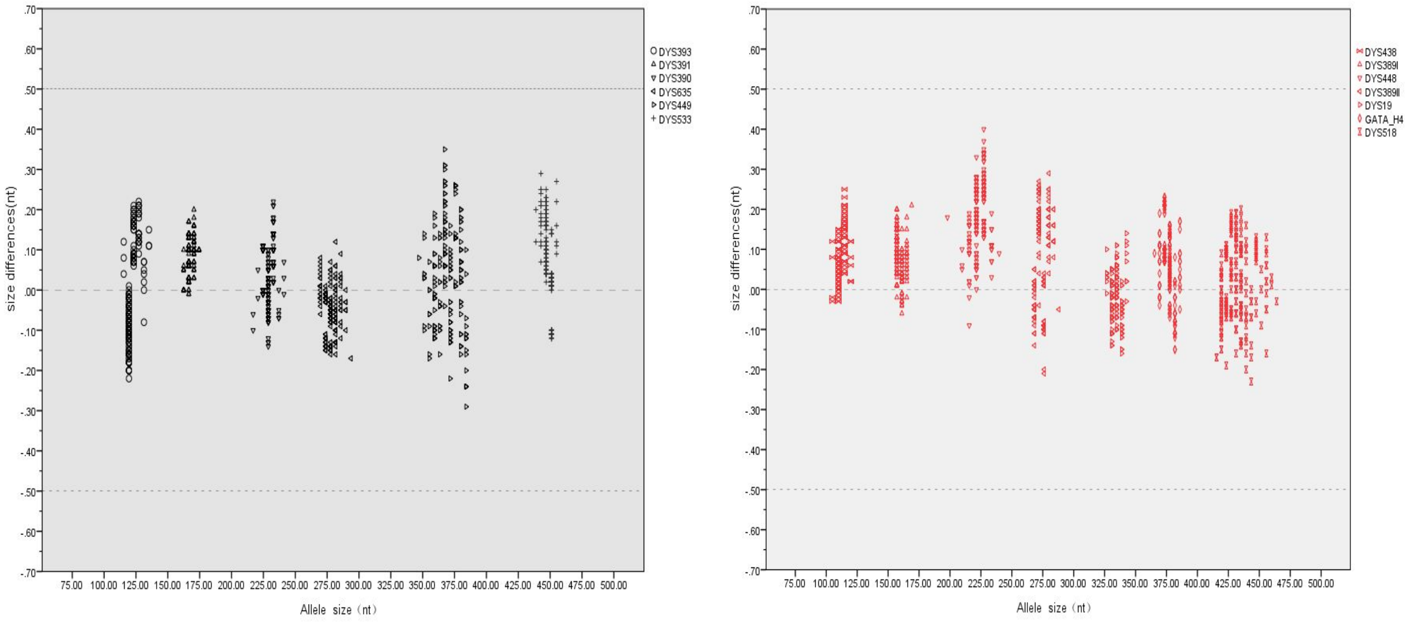

Figure S5. The size deviation of the extracted DNA samples

25 µL system	Control DNA 9948		Control DNA 007	
9948:007	9948 allelesdetected	% complete 9948	007 alleles detected	% complete 007
19:1	27	100%	14	51%
18:2	27	100%	24	88%
16:4	27	100%	27	100%
14:6	27	100%	27	100%
12:8	27	100%	27	100%
10:10	27	100%	27	100%
8:12	27	100%	27	100%
6:14	27	100%	27	100%
4:16	27	100%	27	100%
2:18	21	77%	27	100%
1:19	10	37%	27	100%

Table S2.Detected alleles of the different proportions from the mixture in the 25µL system

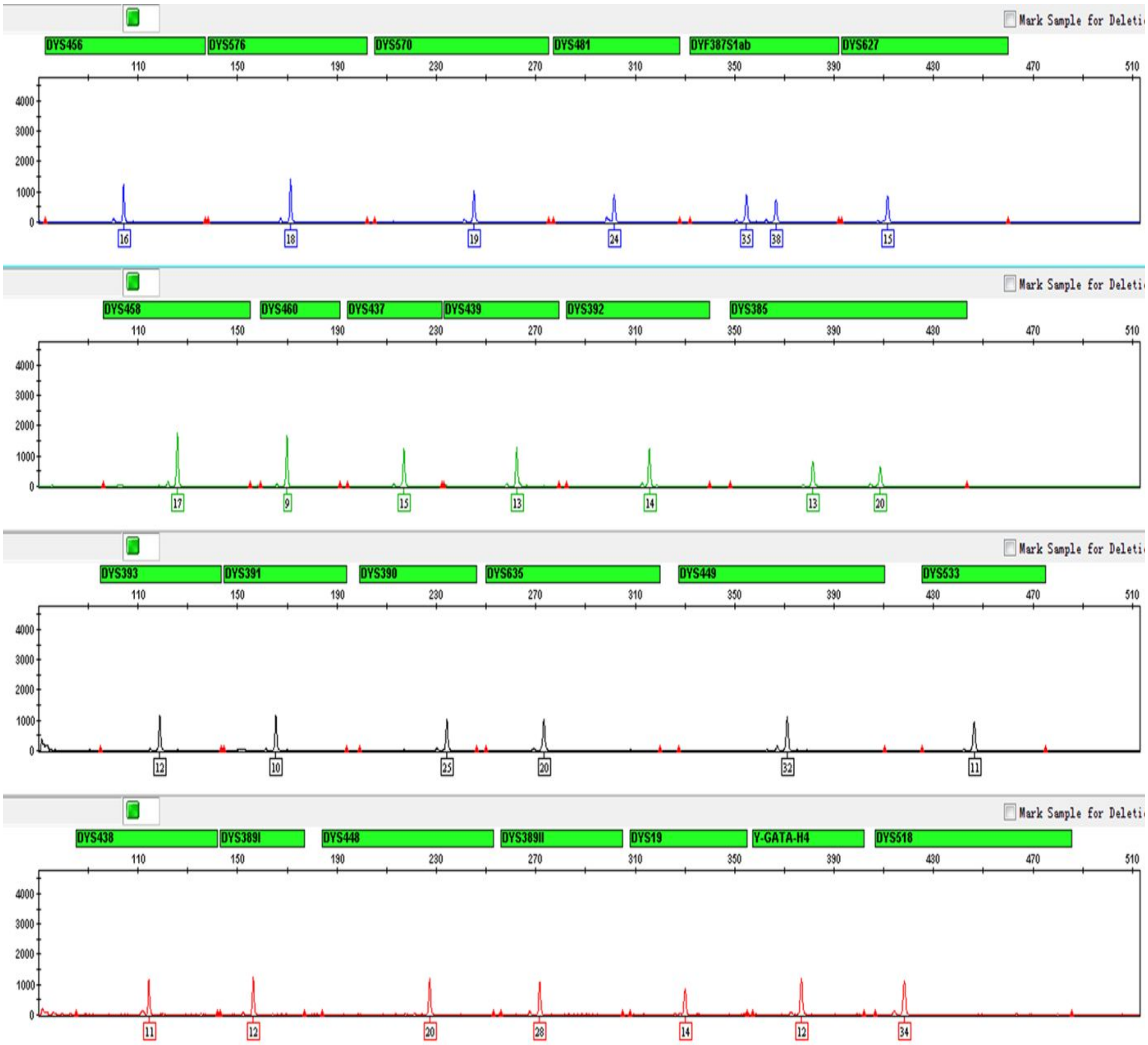

Figure S6. STRtyper-27 electropherograms obtained from one degraded DNA bone sample(0.1ng)
